# Clinical Studies Applying Cytokine-Induced Killer Cells for the Treatment of Renal Cell Carcinoma

**DOI:** 10.1155/2012/473245

**Published:** 2012-11-06

**Authors:** Clara E. Jäkel, Stefan Hauser, Sebastian Rogenhofer, Stefan C. Müller, P. Brossart, Ingo G. H. Schmidt-Wolf

**Affiliations:** ^1^Department of Internal Medicine III, Center for Integrated Oncology (CIO), Universitätsklinikum Bonn, Sigmund Freud Straße 25, 53105 Bonn, Germany; ^2^Department of Urology, Center for Integrated Oncology (CIO), Universitätsklinikum Bonn, Sigmund Freud Straße 25, 53105 Bonn, Germany

## Abstract

Metastatic renal cell carcinoma (RCC) seems to be resistant to conventional chemo- and radiotherapy and the general treatment regimen of cytokine therapy produces only modest responses while inducing severe side effects. Nowadays standard of care is the treatment with VEGF-inhibiting agents or mTOR inhibition; nevertheless, immunotherapy can induce complete remissions and long-term survival in selected patients. Among different adoptive lymphocyte therapies, cytokine-induced killer (CIK) cells have a particularly advantageous profile as these cells are easily available, have a high proliferative rate, and exhibit a high antitumor activity. Here, we reviewed clinical studies applying CIK cells, either alone or with standard therapies, for the treatment of RCC. The adverse events in all studies were mild, transient, and easily controllable. *In vitro* studies revealed an increased antitumor activity of peripheral lymphocytes of participants after CIK cell treatment and CIK cell therapy was able to induce complete clinical responses in RCC patients. The combination of CIK cell therapy and standard therapy was superior to standard therapy alone. These studies suggest that CIK cell immunotherapy is a safe and competent treatment strategy for RCC patients and further studies should investigate different treatment combinations and schedules for optimal application of CIK cells.

## 1. Biology of Renal Cell Carcinoma and Current Treatment Options

Renal cell carcinoma (RCC) accounts for nearly 3% of all adult malignancies. Metastatic RCC has a particularly poor prognosis with an overall survival of 12 months and a 5-year survival of less than 10% [1, 2].

RCC can be divided into three major subtypes with clear cell RCC (70–80%) being the prominent one. Most patients with clear cell RCC carry an inactivated von Hippel Lindau (VHL) tumor suppressor gene. The inactivation of this gene causes an upregulation of several survival and proangiogenic factors such as transforming growth factor-alpha (TGF-*α*) and vascular endothelial growth factor (VEGF).

Apart from clear cell RCC, papillary (10–15%) and chromophobe RCC (5%) are histological subtypes of RCC. These subtypes can be caused, for example, by somatic mutations activating the tyrosine kinase of the cell surface receptor c-MET.

The primary treatment strategy for renal cancer is surgery [3]. Metastatic RCC seems to be resistant to other conventional therapy regimens such as chemotherapy, hormone therapy, or radiotherapy [2, 4]. Until the recent evolution of targeted therapies, interleukin-2 (IL-2) combined with interferon-*α* (INF-*α*) was the first-line treatment strategy for metastatic RCC [3]. Despite substantial toxicity, high-dose IL-2 therapy is superior in terms of response rate compared to low dose IL-2, either given intravenously or subcutaneously [5, 6]. High-dose IL-2 is able to produce durable complete remissions, but still only in a selected group of RCC patients [7, 8]. Subcutaneously injected interleukin-12 (IL-12) is also able to induce tumor regression while being relatively well tolerated [9, 10]. In the study of Gollob and colleagues, IL-12 induced disease regression or stabilization in RCC patients that all had failed prior IL-2 therapy, indicating a different mechanism of action of these two cytokines [9]. Also, the concurrent administration of low-dose IL-2 and IL-12 is well tolerated and able to induce clinical responses [11].

Improved comprehension of the biology of RCC has led to the development and application of new targeted agents. Since 2005, six of these agents have been approved for the treatment of metastatic RCC by the American Food and Drug Administration (FDA) [3]. These drugs act on two distinct pathways to induce tumor regression. Sorafenib, sunitinib, pazopanib, and bevacizumab are angiogenesis inhibitors, acting either on circulating VEGF or on its receptor, in addition axitinib and tivozanib passed phase III studies in RCC. Temsirolimus, a prodrug converted into rapamycin *in vivo*, and everolimus, a rapamycin derivative, are inhibitors of the mammalian target of rapamycin (mTOR) causing a downregulation of mRNAs essential for cell cycle progression [12].

Moreover, adoptive lymphocyte immunotherapies using different activated killer cells have been tested for their efficacy in RCC. Apart from lymphokine-induced killer (LAK) cells and tumor-infiltrating lymphocytes (TILs), cytokine-induced killer (CIK) cells are particularly promising for successful application in RCC patients [13].

## 2. Cytokine-Induced Killer Cells

CIK cells are generated by culturing peripheral blood lymphocytes (PBL) with interferon-*γ* (INF-*γ*) monoclonal antibody against CD3 (anti-CD3) and IL-2 in a particular time schedule. The cytokines INF-*γ* and IL-2 are crucial for the cytotoxicity of the cells and anti-CD3 provides mitogenic signals to T cells for proliferation [14].

Most of these CIK cells (87%) are positive for CD3 and for one of the T-cell coreceptor molecules CD4 (37.4%) or CD8 (64.2%), respectively. IFN-*γ*, added at day 0, activates monocytes providing crucial signals to T cells via interleukin-12 (IL-12) and CD58 (LFA-3) to expand CD56^+^ cells [15]. After 14 days of culture, 37.7% of cells are CD3^+^CD8^+^CD56^+^. These cells are referred to as natural killer T (NK-T) cells and represent the cell type with the greatest cytotoxicity in the CIK cell population [16, 17]. Interestingly, these CD3^+^CD56^+^ double positive CD8^+^ T cells do not derive from the rare CD3^+^CD56^+^ cells in the starting culture but from proliferating CD3^+^CD8^+^CD56^−^ T cells [18]. Their cytotoxicity is nonmajor histocompatibility-complex (MHC)-restricted and they are able to lyse a variety of solid and hematologic tumors [15, 19]. Cell lysis is not mediated through FasL but through perforin release [20]. CIK cell cytotoxicity depends on NKG2D recognition and signaling. Here, an important factor is the addition of high dose IL-2 during culturing as it is critical for the expression of the NKG2D adapter protein DAP10: T cells activated with only low concentrations of IL-2 showed upregulated NKG2D but no DAP10 expression and were not capable of cytolysis [21]. Accordingly, several studies have revealed the expression of NKG2D ligands (e.g., MICA, MICB, ULBP 1–4) on both solid and hematologic tumors [22–24].

Altogether, CIK cells are very effective cytotoxic cells which have a great potential to play a major role in cancer therapy. Advantages of these cells are their high cytotoxicity even at low cell numbers, their high proliferative rate, their non-MHC-restricted cytotoxicity, and their activity against multidrug resistant tumor cell lines [14, 15, 25].

**Figure 1:**
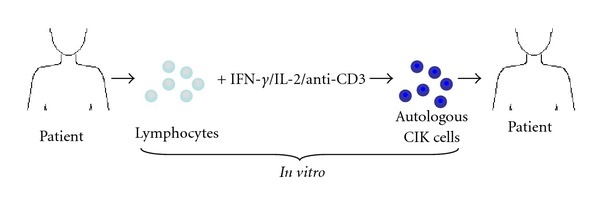
Generation and clinical application of CIK cells. Lymphocytes are extracted from the patients' blood and stimulated with different cytokines. The resulting CIK cells are re-infused into the patient. IFN-*γ*: interferon-*γ*; IL-2: interleukin-2; anti-CD3: anti-human CD3 monoclonal antibody.

For clinical application of autologous CIK cells whole blood is drawn from the patient and lymphocytes are isolated. These cells are then stimulated *in vitro* with INF-*γ*, IL-2 and anti-CD3 for at least fourteen days to generate CIK cells. Finally, the CIK cells are reinfused into the patient (Figure 1).

LAK cells, generated by culturing blood lymphocytes with high dose IL-2, are also able to lyse tumor cells in a non-MHC-restricted manner and have been applied *in vivo* for the treatment of various tumors [26–29]. However, CIK cells can be obtained more easily and revealed a higher cytotoxic activity against tumor cells [14, 25, 30, 31]. In a study of Lu and Negrin (1994), the antitumor effects of CIK and LAK cells have been compared in lymphoma bearing SCID mice and CIK cells were shown to be more potent in the specific killing of tumor cells [32].

Another T-cell-based approach for immunotherapy in cancer includes TILs, which can be directly isolated from tumor tissue and expanded *in vitro* with IL-2. These cytotoxic cells possess a higher antitumor activity than LAK cells [33, 34]. Still, it is difficult to recover suitable numbers of these cells for therapeutic approaches. Moreover, the adoptive therapy of cancer with TILs is hampered by several factors such as resistance of tumor cells to the apoptotic pathway mediated by TILs, the weak definition of target antigen expressed on tumor cells, and the poor localization of these cells to the tumor side [35, 36].

## 3. Clinical Studies on CIK Cells for the Treatment of RCC

The first clinical study applying autologous CIK cells for cancer therapy was performed by Schmidt-Wolf and colleagues in 1999 [37]. In this study, autologous CIK cells were transfected with the IL-2 gene and re-infused into the participants—one patient with renal cancer, seven patients with colorectal cancer, and two patients with lymphoma. At the time of entry into the study the last conventional treatment was more than 28 days ago. The treatment schedule for the study consisted of one cycle of five infusions of transfected CIK cells and, after three weeks, a second cycle of five either transfected or untransfected CIK cell infusions. 

IL-2-transfected CIK cells were detectable in the patients' blood for up to two weeks after treatment and the cytotoxicity of peripheral blood mononuclear cells (PBMC) increased during treatment. Moreover, elevated serum levels of transforming growth factor-*β* (TGF-*β*), IFN-*γ*, and granulocyte macrophage colony-stimulating factor (GM-CSF) were measured.

Adverse events were only transient, with three patients with grade 2 fever and one patient with anemia. At the end of the study, one patient had a complete response (CR) with disappearance of the tumor for at least four weeks; three patients showed no change by treatment (stable disease), and six patients remained in progressive disease (PD). 

Among 66 patients with solid tumors, six patients with RCC were treated with multicycles of autologous CIK cells in the study of Ren et al. from 2006 [31]. Untreated PBMC and CIK cells of 22 randomly selected patients were collected for phenotyping. A significant increase in total CD3^+^, CD4^+^, CD8^+^, CD25^+^ and CD3^+^CD56^+^ cells was demonstrated in the CIK cell cultures. 

Patients received two intravenous infusions of autologous CIK cells per cycle in a one-day interval and twenty patients were given three or more cycles in one-month intervals. No serious adverse events occurred during treatment. After several cycles of CIK cell therapy, the patients' PBMC showed significant increases in the cytotoxicity towards K562 leukemia cells and the secretion of IFN-*γ*, IL-2, and TNF-*α*.

In conclusion, neither the clinical stage of the patients, nor the number of administered cells or the proportions of CD4^+^ and CD8^+^ T cells had an influence on the effectivity of CIK cell therapy. However, there was a clear relationship between the clinical outcome and the number of cycles given and the proportion of CD3^+^CD56^+^ cells in the CIK cell population, respectively.

In 2009, Olioso and colleagues conducted a CIK cell therapy trial with twelve patients with advanced solid tumors including five patients with metastatic RCC [38]. Phenotypic analysis showed that the starting CIK cell cultures of the patients contained a median of 66% CD3^+^ and 4% CD3^+^CD56^+^ cells, which increased to 97% CD3^+^ and 30% CD3^+^CD56^+^ after 21 days. 

The patients were treated with three cycles of autologous CIK cell infusions followed by a three-week rest. After the first three infusions, the clinical response was evaluated, and, in case no tumor progression was observed, the patients received three further cycles of CIK cell infusions. RCC patients additionally received low doses (2-3 × 10^6^ IU/m^2^/day) of subcutaneous recombinant human IL-2 (rhIL-2) five times weekly. 

All side effects were transient, easily controllable, and a dose-limiting toxicity was not reached; two patients developed low grade fever and/or chills and the RCC patients additionally treated with IL-2 developed grade 2 fever without decrease in performance status. 

Response evaluation was classified according to the Response Evaluation Criteria in Solid Tumors (RECIST criteria) [39]. At the end of the study, three patients were withdrawn from the study; three patients (33%), including one RCC patient, had no evidence of tumor for at least six months (CR), two of them had additionally been treated with IL-2; two patients, both RCC patients, had stable disease (SD) without new lesions and neither a 50% reduction (partial response, PR) or 25% increase (PD) in tumor size. There was no significant relationship between the number of infused CIK cells and the clinical response but, remarkably, an increased production of IFN-*γ* and TNF-*α* was detected in responding patients but not in nonresponders. After a median followup of 34 months, five patients (56%) were still alive, among them three patients with RCC.

The CIK cell study of Su et al. (2010) exclusively included patients with metastatic RCC [40]. The treatment schedule involved two to three cycles of CIK cell infusions followed by a three-week rest. 

The CIK cells of all sixteen patients were tested for their *in vitro* toxicity against K-562 NK-sensitive leukemia cells, 293 transformed embryonic kidney epithelial cells and SK-RC-42 RCC cells at an effector-to-target (E/T) ratio of 60 : 1. The median toxicity was 77.2%, 50.4%, and 32.1%, respectively. Phenotypic analysis of the CIK cells generated *in vitro* showed an increase in CD3^+^, CD4^+^, CD8^+^, CD3^+^CD56^+^ and NKG2D^+^ cells. Furthermore, a significant decrease in CD4^+^CD25^+^CD127 low^+^ regulatory T (Treg) cells was determined. Although to a lesser degree, similar changes were also detected in PBMC before and fourteen days after CIK cell treatment.

The treatment was well tolerated with only transient and controllable adverse events. Three patients had a CR, that is, disappearance of the tumor for at least four weeks, and one patient had a PR (>30% decrease in tumor size) giving a response rate of 25%. Moreover, six patients achieved SD with neither a >20% increase nor a >30% decrease in tumor size. As in the study of Olioso et al., an increased production of IFN-*γ* and TNF-*α* by PBMC after CIK cell treatment was detected. Again, this increase correlated with the clinical response.

Recently, the largest study of autologous CIK cell immunotherapy in metastatic RCC so far was published by Liu and colleagues [13]. The prospectively randomized study included 148 patients treated either with CIK cell infusions (arm 1, *n* = 74) or with IL-2/IFN-*α* therapy (arm 2, *n* = 74). In arm 1, one treatment cycle consisted of CIK cell amplification from day one to fourteen, CIK cell infusions on days fifteen and sixteen and rest until day 30; one cycle was conducted every month and a median of ten cycles was given. The patients in arm 2 were treated with subcutaneous IL-2 and IFN-*α* on day one, three and five in week one to four and then at rest for week five and six. One cycle was performed every six weeks with a median of 2.5 cycles.

As in the study of Su et al., cytotoxicity assays were performed with CIK cells. The cytotoxicity against the SK-RC-42 cell line at an E/T ratio of 60 : 1 was almost the same with 32.2%. The cytotoxicity against the RCC cell line 786-O was 35.4%.

Adverse events were much more frequent and serious in patients who received cytokine therapy while patients treated with CIK cells developed only transient fever, chills, fatigue, headache, and anemia.

Tumor evaluation was performed two months after start of treatment and scored according to RECIST criteria [39]. Among the patients who received CIK cell infusions, thirteen patients had a CR (18%) and 26 patients had a PR (35%) giving an overall response rate of 53%; 25 patients achieved SD (34%) and 10 patients remained in PD (14%). The overall response rate in Arm 2 (IL-2/IFN-*α*) was significantly lower with 27% (5 patients with CR (7%), fifteen patients with PR (20%)); 25 patients achieved disease stabilization (34%) and 29 patients remained in disease progression (39%). Also the progression-free survival and overall survival rates were significantly higher in patients who received CIK cells.

In conclusion, CIK cell treatment significantly improved the prognosis of metastatic RCC. Moreover, the prognosis of patients who received at least seven cycles of CIK cell infusions was considerably better than the prognosis of patients who received less than seven cycles. 

From January 2002 to June 2006, Lei and colleagues examined the effect of CIK cell therapy applied along with chemo- and cytokine (IL-2/IFN-*α*-2b) therapy [41]. The therapy schedule started 30 to 60 days after tumor nephrectomy. Patient group A (*n* = 18) received several cycles of CIK cell treatment combined with cycles of cytokine and chemotherapy. The ten patients in the second study group were administered several cycles of cytokine and chemotherapy only.

In group A (CIK cell therapy), three patients died within two years and fifteen were still alive and at good health at the end of the study. Six patients in group B died within 3 years and 4 were still alive and at good health at the end of the study.

All in all, the authors concluded that the best period for CIK cell infusions is between two cycles of chemo-/radiotherapy and CIK cell therapy has a good effect on post-operative RCC patients.

Similarly, Li et al. experienced CIK cell therapy as a safe and effective therapy for localized renal carcinoma after radical nephrectomy [42]. Fifteen days after nephrectomy, eight patients were treated with CIK cells while another four patients were given biotherapy. After followup between one and four years, six patients who had received CIK cells had a CR and one patient a PR (according to RECIST criteria [39]). Within the control group, all patients had metastases within two to fifteen months after nephrectomy. 

In 2006, Wang et al. conducted a CIK cell therapy trial additionally applying an autologous tumor cell lysate-loaded dendritic cell (DC) vaccine [43]. They enrolled ten patients with advanced RCC who already had a complete excision of the ill kidney. They received a minimum of eight weekly intradermal DC vaccinations and four biweekly CIK cell infusions. After a median followup of eleven months, one patient had a PR, six patients had SD and two had PD, one patient was lost. In conclusion, the side-effects of the CIK cell infusions were tolerable and transient and the applied CIK cells were able to induce increases in the levels of CD3^+^, CD4^+^, CD4^+^CD8^+^, and CD56^+^ cells after two months of treatment.

The results of the phenotypic analyses and cytotoxicity assays conducted with CIK cells from patients are depicted in Table 1. The study design, clinical results, and conclusions of all studies discussed above are outlined in Table 2. 

## 4. Summary and Future Prospects

The clinical studies discussed above establish CIK cell immunotherapy as a safe and valuable treatment strategy for RCC patients, even in advanced disease stages. CIK cell application, either alone or together with bio- or chemotherapy, was able to induce complete responses in RCC patients. Complete response is defined by the NCI as the disappearance of all signs of cancer in response to treatment. Actually, CIK cells applied alone or along with standard therapies were either way superior compared to the standard therapies [13, 41]. In the study of Liu et al., patients treated with CIK cell therapy had a higher response rate, a longer progressive-free survival, and a longer overall survival than patients who received standard cytokine therapy [13].

Importantly, also the toxicity profile of CIK cell application was favorable in all studies. Adverse events of CIK cell infusions were only mild, transient, and easily controllable. In none of the clinical studies a dose-limiting toxicity was reached. Regarding all therapy schedules and results, the application of several CIK cell infusion cycles seems to be critical for the clinical outcome. Still, the interaction of CIK cell therapy with conventional therapies like chemotherapy or IL-2/IFN-*α* biotherapy should be investigated further to eventually benefit from both treatment options. Moreover, the synergy between CIK cells and targeted therapies applied in RCC has not been investigated and should be subject of future research.

In several studies the CIK phenotype and cytotoxicity were analyzed. CIK cell populations showed significant increases in CD3^+^, CD4^+^, CD8^+^, and CD3^+^CD56^+^ and a decrease in Treg cells during *in vitro* expansion [31, 38, 40]. After CIK cell infusions, these shifts could also be detected *in vivo* [40]. Several cytotoxicity assays were performed with different target cells such as the NK-sensitive leukemia cell line K562 and RCC cell lines. At E/T ratios between 20 : 1 and 60 : 1, the cytotoxicity of CIK cells ranged from 32% to 77% [13, 38, 40]. Interestingly, also the cytotoxic activity of PBMC and their production of TNF-*α* and IFN-*γ* increased during treatment [31, 37, 38, 40]. 

Attempts to decrease the amount of Treg cells within the CIK cell population are promising to further develop the cytotoxicity profile of CIK cells. Treg cells are known to inhibit cytotoxic T-cell responses and, in fact, it was demonstrated that the depletion of Treg cells before culturing of CIK cells could significantly increase the cytotoxicity of CIK cells [44]. Similarly, the addition of IL-6 to the CIK cell culture was shown to decrease the fraction of Treg cells and increase the cytotoxicity of CIK cells *in vitro* [45]. 

The availability of large amounts of CIK cells and their non-MHC-restricted tumor targeting make these cells a promising tool for immunotherapy for RCC. The studies discussed here demonstrate the safety of CIK cell immunotherapy, showing low systemic toxicity while indicating clinical activity. Unfortunately, the variations in methods and clinical evaluation between the studies hamper definite conclusions about the clinical efficacy of CIK cell therapy and more studies are needed to elucidate the best treatment schedule for CIK cell therapy in RCC patients. Recently, the International Registry on CIK cells (IRCC) was created, aiming at collecting data and setting a new standard on the report of results from clinical trials with CIK cells [46]. Currently, a study to evaluate the clinical efficacy of DC-activated CIK cells in RCC patients following conventional therapy is ongoing (NCT01240005) [47].

## Figures and Tables

**Table 1:** Summary of *in vitro* assays conducted with CIK cells of study participants.

Study	Phenotypic analysis of CIK cells	Cytotoxicity assays
Schmidt-Wolf et al. [37]	—	Cytotoxic activity of PBMC against HLA-matched carcinoma cell lines and K562 cells increased during treatment
Ren et al. [31]	Significant increase in CD3^+^, CD4^+^, CD8^+^, CD25^+^, and CD3^+^CD56^+^ cells after 14–16 days of culture	Cytotoxicity of PBMC against K562 cells after multicycles of CIK cell infusions significantly increased
Olioso et al. [38]	After 21 days of culture, CD3^+^ cells expanded 34-fold and CD3^+^CD56^+^ 270-fold; increases in CD3^+^, CD8^+^, and CD3^+^CD56^+^ cells in circulating lymphocytes seven days after CIK cell infusion	Cytotoxicity of CIK cells from RCC patients tested against 293 cells: at an E/T of 20 : 1, the median percentage lysis was 45%; at an E/T of 40 : 1, 54% were lysed
Su et al. [40]	After 14 days of culture, increases in CD3^+^, CD4^+^, CD8^+^, CD^+^CD56^+^ and NKG2D^+^ cells were detected while the number of CD4^+^CD25^+^CD127 low^+^ (Treg) cells decreased; the same changes were detected in PBMC after CIK therapy	Cytotoxicity of CIK cells tested against K562 cells (E/T ratio of 60 : 1): the median toxicity was 77, 2%; cytotoxicity of CIK cells tested against RCC cells (E/T ratio of 60 : 1): CIK cells lysed 50,4% of 293 cells and 32,1% of SK-RC-42 cells
Liu et al. [13]	—	Cytotoxicity of CIK cells tested against RCC cells (E/T ratio of 50 : 1): CIK cells lysed 35,41% of 786-O cells and 32,17% of SK-RC-42 cells
Wang et al. [43]	After treatment for two months, levels of CD3^+^, CD4^+^, CD4^+^CD8^+^, and CD56^+^ increased significantly	—

PBMC: peripheral blood mononuclear cells; K562 cells: NK-sensitive leukemia cells; 293 cells: embryonic kidney epithelial cells; E/T ratio: effector-to-target ratio; SK-RC-42 cells: renal cell carcinoma cells; 786-O cells: renal cell carcinoma cells.

**Table 2:** Clinical studies applying CIK cells for the treatment of RCC.

Study	Number of patients	Therapeutic approach	Clinical response	Conclusion
Schmidt-Wolf et al. [37]	10 (1 RCC)	Auto-CIKs transfected with IL-2 gene	1 CR, 3 SD, 6 PD	Low toxicity of CIK cell therapy; CR in RCC patient
Ren et al. [31]	66 (6 RCC)	Auto-CIKs	40 SD, 3 PR, 11^†^, 12 lost	Disease stage had no influence on antitumor activity of CIK cells; number of infusion cycles and proportion of CD3^+^CD56^+^ cells important for clinical outcome
Olioso et al. [38]	12 (5 RCC)	Auto-CIKs	3 CR (1 RCC), 2 SD (both RCC), 3 withdrawn from study; response rate: 33%	No significant differences in number of infused CIK cells between responders and nonresponders; 2 of 3 CR received additional IL-2/IFN-*α* therapy
Su et al. [40]	16 (all RCC)	Auto-CIKs	3 CR, 1 PR, 6 SD, 6 PD; response rate: 25%	AE transient and controllable; increased production of IFN-*γ* and TNF-*α* by PBMC after CIK cell treatment
Liu et al. [13]	148 (all RCC)	*Arm 1:* auto-CIKs *Arm 2:* IL-2 + IFN-*α*	*Arm 1:* 13 CR, 26 PR, 25 SD, 10 PD; response rate: 53% *Arm 2:* 5 CR, 15 PR, 25 SD, 29 PD; response rate: 27%	CIK cell treatment significantly improves prognosis of metastatic RCC; prognosis significantly better in patients who received ≥7 cycles of CIK infusions
Lei et al. [41]	28 (all RCC)	*Group A:* auto-CIKs + IL-2 + IFN-*α*2b + chemo *Group B:* IL-2 + IFN-*α*2b + chemo	January 2002–June 2006: *Group A:* 15 at good health, 3^†^ *Group B:* 4 at good health, 6^†^	Best period for infusions between 2 cycles of chemo/radiotherapy; CIK cells had a positive effect on postoperative RCC patients
Li et al. [42]	12 (all RCC)	*Group 1:* auto-CIKs *Group 2:* biotherapy	*Group 1:* 6 CR, 1 PR *Group 2:* All PD	CIK therapy safe and effective for localized RCC patients after radical nephrectomy
Wang et al. [43]	10 (all RCC)	Auto-CIKs + autologous renal tumor lysate-loaded DCs	1 PR, 6 SD, 2 PD, 1 lost	AE tolerable; short-term efficacy on advanced RCC through induction of specific antitumor immunity

Auto-CIKs: autologous CIK cells; IL-2: interleukin-2; CR: complete response; SD: stable disease; PD: progressive disease; PR: partial response; IFN-*α*: interferon-*α*; AE: adverse events; IFN-*γ*: interferon-*γ*; TNF-*α*: tumor necrosis factor-*α*; PBMC: peripheral blood mononuclear cells; chemo: chemotherapy; ^†^dead; DCs: dendritic cells.
